# U-Th dated speleothem recorded geomagnetic excursions in the Lower Brunhes

**DOI:** 10.1038/s41598-018-38350-4

**Published:** 2019-02-04

**Authors:** Jean-Pierre Pozzi, Louis Rousseau, Christophe Falguères, Geoffroy Mahieux, Pierre Deschamps, Qingfeng Shao, Djemâa Kachi, Jean-Jacques Bahain, Carlo Tozzi

**Affiliations:** 1 Ecole Normale Supérieure, Laboratoire de Géologie UMR CNRS 8538 PSL, Paris, 75005 France; 2 MNHN, Département Homme et Environnement UMR 7194 HNHP, Paris, 75013 France; 3 UPJV, EA 7511 Bassins Réservoirs Ressources, Amiens, 80000 France; 4 IRD CEREGE, Géologie et géochronologie UMR 161, Aix en Provence, 13545 France; 5 Nanjing Normal University, School of Geographical Sciences, Nanjing, 210023 China; 6 UPJV, Modélisation Information and Systèmes EA 4290, Amiens, 80000 France; 7 Universita di Pisa, Dipartimento di Scienze Archeologiche, Pisa, 56126 Italy

## Abstract

The study of geomagnetic excursions is key for understanding the behavior of the magnetic field of the Earth. In this paper, we present the geomagnetic record in a 2.29-m-long continuous core sampled in a flowstone in Liguria (Italy) and dated to the Lower Brunhes. The cored flowstone developed from Marine Isotopic Stage (MIS) 13 to MIS 7, according to 21 U-series dates. The mean growth rate is closely related to glacial and interglacial isotopic stages. Magnetic remanence was measured using u-channel and deconvolved. Four geomagnetic excursions were recorded at the same location, in a single flowstone, during interglacial MIS 11 and 13; Basura 1, 2, 3 and 4, at depths of 213 cm, 181, 160 and 92 cm, respectively. Due to the uncertainties of U-Th dating, the timing of the three events, namely Basura 1, 2 and 3 overlaps. The Basura 4 is well-dated to 417 + −7/8 ka and is clearly distinguishable from the others. It should therefore be considered as a possible excursion.

## Introduction

Geomagnetic excursions are short worldwide episodes of intermediate polarity of the Earth’s magnetic field^1^, beyond the range of secular variation^2–5^. Although they are recorded in almost all types of rocks, the inventory of geomagnetic excursions in the Lower Brunhes, before 200 ka, has not yet been clearly established. An accurate chronology and duration of geomagnetic excursions is important for understanding diverse aspects of Quaternary geology, including the recognition of astronomical events, paleontological and anthropological stratigraphic markers^6^. The acquisition of magnetization in sediments, where sedimentation is continuous, is not instantaneous and depends on the thickness of the lock-in zone. Radiometric and astronomical dating methods have been used to date these sediments. In the case of lava sequences, the acquisition of magnetization is instantaneous, but depends on the episodic occurence of eruptions. The construction of a reliable inventory of excursions thus depends on the convergence of lava, sediment and speleothem records with their independent dating methods.

Advances in paleomagnetism and dating techniques applied to speleothems have led to increased interest in their potential as geomagnetic archives^7–11^ and both geomagnetic and climatic archives^12,13^. The use of speleothems as geomagnetic records presents key advantages over sedimentary records from lake and marine sediments or eolian loess deposits, including nearly instantaneous magnetization lock-in, which preserves the paleomagnetic signal at the time of calcite deposition. The principal remanence carrier is magnetite^11,14,15^. Speleothems can be dated with very high precision using U-series dating, namely ^230^Th/U^16–18^ allowing determination of mean growth rate between dated intervals.

To our knowledge, only two articles have unambiguously reported geomagnetic excursions in radiometrically-dated speleothems. The Blake excursion was dated between 116.5 ± 0.7 ka and 112 ± 1.9 ka^19^, and the Laschamp excursion was dated between 42.25 ka and 39.70 ka (center age 41.01 ± 0.35 ka^20^). The dates of the Laschamp and Blake excursions give low 2σ uncertainties using the U-Th method in this dating range.

In this paper, for the first time, we provide paleomagnetic evidence of the recording of Lower Brunhes geomagnetic excursions in a speleothem from Liguria (Italy). The studied record is a 2.29-m-long continuous core, drilled from a calcitic flowstone which grew on the cave floor, the growth record of which has been was constrained by twenty-one U-Th dates.

## Research Setting

**Figure 1:**
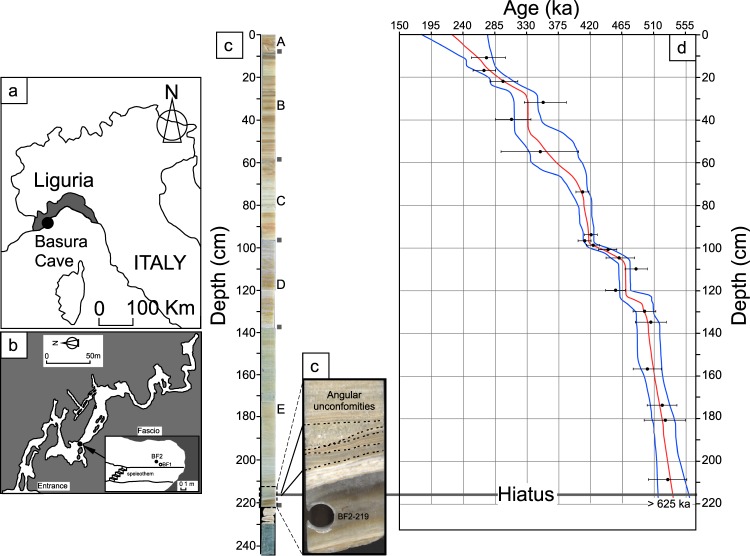
(**a**) Location of Liguria (Italy). (**b**) Schematic map of Basura Cave showing the position of the flowstone with respect to the entrance of the cave and enlarged view of the sampling site showing the relative position of the BF1 and BF2 cores. Square symbols show the limit of the five sections. (**c**) General photograph of the BF2 core showing how the calcite laminae change with depth. Square symbols show the limit of the five sections. Enlarged view near the base of the core shows, below 216 cm, a ∼1.5-cm-thick zone displaying disturbed growth with a succession of angular unconformities. d- U-Th dates (Supplementary Table 1) and age model of BF2 core. Dotted curves give the 2σ confidence interval. Below the hiatus, it is dated to older than 625 ka.

Basura Cave is located in Liguria, Italy (N44^◦^07′ E8^◦^12′; Fig. 1a), in the Triassic limestone of Monte San Pietro (Briançon series), which rises to 891 m above sea level. The area contains abundant Paleolithic sites recording evidence of human occupation during the Middle and Upper Pleistocene^21,22^.

Basura Cave is part of a large karst system extending over at least 4 km on several levels. In the Fascio chamber of the cave, (Fig. 1b), a 2.30-m-thick flowstone extends laterally over a surface of about 100 m^2^. At the sampling site, the thickness of limestone above the cave is estimated to be 50 m. The altitude of the sampling site is 8 m above the cave entrance. The sampling site is located 200 m from the narrow cave opening (Fig. 1b). Therefore, it is unlikely that the speleothem collected many airborne particles. No fossils or pollen were detected.

During previous work^23^, a first paleomagnetic study of the flowstone was performed on a core named Basura Fascio 1 (BF1), dated by the electron spin resonance (ESR) method. The paleomagnetic directions were retrieved using a spinner magnetometer and a few standard samples allowing for the recognition of a polarity reversal area at the basal part of the flowstone. In order to conduct a more precise and continuous analysis of the speleothem, we decided to take a new core from the same flowstone. The core, referred to as Basura Fascio 2 (BF 2), was drilled a meter away from the BF1 drilling location (Fig. 1b). The core is 2.29 m long with a diameter of 4 cm. The core was drilled vertically in five sections (Fig. 1c), each with a maximal length of 90 cm. The BF2 core was not oriented *in situ* but the five adjacent sections are continuous and BF2 was oriented with respect to the North using the normally magnetized A and B sections. The core was cut in half along the vertical axis perpendicular to the growth layers, in order to use the first half of the core for paleomagnetic measurements and dating, and the second half for mineralogy and rock magnetism. The first half-core was re-sliced for u-channel measurements.

The axial cross-section of BF2 (Fig. 1c) reveals a structure formed by a horizontal succession of millimetric to centimetric-scale light brown to grey and white calcitic laminae. The core shows an angular unconformity of 18^◦^ at 216 cm. Below the unconformity, a 1.5 cm-thick zone displays disturbed growth with a succession of angular unconformities representing a temporal discontinuity or hiatus; the only one identified so far. This tilt could be of tectonic origin or result from fluid flow changing direction due to the evolving topography of the base of the cave.

## Methods

### U-series dating method

A first set of seven U-series dates was measured by thermal ionization mass spectrometry (TIMS) at the CEREGE (Aix-en Provence, France) and GEOTOP (Montreal, Canada), using two different ^236^U^−233^U^−229^Th mixed spikes. These spikes were calibrated following the procedure described by Deschamps^24^ (Supplementary Methods).

A second set of 14 U-series dates was performed at Nanjing Normal University (China). The procedures used for U-Th chemical separation and isotopic measurements are detailed in Shao^25^ (Supplementary Methods).

Age data were processed using the StalAge algorithm, specifically designed for speleothem age models^26^.

### Scanning electron microscopy method

Fresh rock fragments were observed and scanned under a ZEISS ZIGMA SEM microscope coupled to an Oxford Instrument X-MAX EDS detector at the Ecole Normale Supérieure in Paris (France). The accelerating voltage is 15 keV (LaB6 field emission gun). The width of the spot is ∼1 μm. Quantitative compositional analysis is provided by energy dispersive spectra (EDS) using the INCA Software (Oxford Instruments).

### Rock magnetic methods

#### Hysteresis

Hysteresis curves and first-order reversal curves (FORC) were carried out using a vibrating sample magnetometer (VSM) from Princeton Measurements Corporation. Since the magnetization of speleothems is very weak, each sample was crushed into powder and tightly packed into a medicinal capsule, in order to maximize the amount of material for the measurement, instead of using a millimetric chip fixed by grease, as is usual with the VSM. FORC diagrams were processed using the FORCinel software^27^ and the VARIFORC option^28^.

#### High and low temperature

Thermal variation of magnetic susceptibility was measured using AGICO KLY3S (sensitivity ∼10^−7^ SI), in an argon-controlled atmosphere and using steps of 9 °C. The low field magnetic susceptibility was too weak to be measured on the core. Low temperature analyses were carried out using the Quantum Design Magnetic Properties Measurement System (MPMS). 2.5 T saturation IRM (SIRM) was imparted at room temperature (RT-SIRM) and the specimen cooled from 300 K to 10 K (5.3 K to 4 K steps). For LT-SIRM, a 2.5 T IRM was imparted at 10 K (LT) after zero-field cooling (ZFC) and was thermally demagnetized by warming to 300 K (9 K to 1 K steps).

The rock magnetic measurements were conducted at the Institut de Physique du Globe de Paris-Institut de Minéralogie, de Physique des Matériaux et de Cosmochimie Mineral Magnetism Analytical Facility.

### NRM measurement method

The natural remanent magnetization (NRM) of the core was measured using a 2 G Enterprise DC-Squid Superconducting Magnetometer (SRM) at the CEREGE (Aix en Provence, France), with noise level to the order of 10^−6^ A/m and at the Ecole Normale Supérieure in Paris (ENS), using a 2 G Enterprise 755 (SRM). The characteristic remanence components (ChRM) were retrieved by means of AF demagnetization using the online 2G system. After initial NRM measurement, stepwise AF demagnetization was carried out in 2.5 mT increments in the 2.5–35 mT interval and 5 mT increments in the 35–90 mT interval. Measurements of remanence, and alternating field demagnetization were carried out using u-channel with 1-cm-step. After the demagnetization of these samples, anhysteretic remanent magnetization (ARM) was produced by the combination of a 50 mT alternating magnetic field and a 0.5 mT direct magnetic field, both conducted along the vertical axis of the core. The magnetic measurements were made over the whole length of the 229 cm core but data were only processed up to 221 cm. Deconvolution was performed using graphical UDECON software^29^.

The demagnetization curves were analyzed using DAEI software^30^, where all the standard demagnetization diagrams (orthogonal projection diagrams, stereographic projection of unit vectors, variation of remanence intensity during stepwise demagnetization) are featured. The ChRM and the Maximal Angular Deviation (MAD) are computed by principal component analysis (PCA) at selected intervals of demagnetization steps, following the Kirschvink method^31^.

## Results

### Age model

The age model was based on twenty-one U-series dates summarized in Supplementary Table 1 (Fig. 1d). A hiatus at 219 cm of depth is inferred by the age difference between 216 cm, dated to 536 ± 25/18 ka and 219 cm, dated to older than 625 ka (Fig. 1d). From 216 cm to the top, the core grew continuously from marine isotopic stage (MIS) 13 (533-478 ka) to MIS 8 (300-243 ka)^32^.

The flowstone stopped growing near 225 ka, at the beginning of MIS 7 (191–243 ka). The age model shows significant changes in the growth rate.

### Scanning electron microscopy

An example of a scanning electron microscopy photograph of the BF2 flowstone is shown in Supplementary Fig. 2. More generally, composition mapping and EDS spectra show Ti-poor and Ti-rich iron oxides, mainly represented by small crystals with grain size up to 10 μm and probably of allochthonous origin. These grains were probably demagnetized quickly and therefore do not contribute to ChRM. In the background, many small grains with a size in the order of 1 μm revealed iron oxide which contributes to ChRM.

### Rock magnetic results

#### Hysteresis and FORC results

Hysteresis curves were constructed from 16 samples in the 52 cm to 90 cm depth interval and from 185 cm to 210 cm. A small ferromagnetic contribution remains in all samples with a mean saturation field of ∼0.3 T, compatible with magnetite (see Supplementary Fig. 3a). The hysteresis data are plotted on the theoretical unmixing Dunlop diagram^33^ (see Supplementary Fig. 3b). The data are concentrated in a small region: 0.15< Mrs/Ms <0.40 and 1.5< Hcr/Hc <3. The representative points follow the general trend of the single-domain + multi-domain model (SD + MD) mixing curves, most of which are slightly above the SD + MD mixing curves and not far below the SD + 10 nm SP (super paramagnetic) mixing curves. It seems reasonable to assume that this represents broad magnetic size distribution through the single-domain (SD) and pseudo-single-domain (PSD).

After treatment by the Cumulative Log-Gaussian function^34,35^, the best matches with the raw isothermal remanent magnetization (IRM) curves are obtained by considering two components characterized by a bimodal association of coercivity (see Supplementary Fig. 3c), namely, a low coercive phase (component 1), thought to be magnetite/maghemite (B_1/2_ ∼24 mT) and a higher coercive phase (components 2) with B_1/2_ ∼510 mT, in the range of hematite or goethite. Component 2 has low remanence intensity, therefore we have not considered this component in our interpretation. Mean acquisition fields (i.e., B_1/2_ ∼Hcr) and the dispersion parameter (DP ∼0.28) are similar to those of the magnetite contained in speleothems^14^. Magnetite has been identified as a major magnetic mineral in speleothems, often associated with maghemite^14,15,36^. Maghemite, produced by progressive magnetite oxidation, also shows a comparable range of coercivity^11^.

The FORC diagram^37–39^ is represented for VARIFORC (SF min = 3) and for SF = 3 (see Supplementary Fig. 3d,e), for a sample in the lower part of the core at a depth of 212 cm, where magnetization is not too low. The saturating field was set to 1 T and the averaging time was 0.1 s. The hypothesis of two components, a pseudo-single-domain and a single-domain, is visible. This conclusion corroborates the result inferred from the hysteresis plot.

Hysteresis measurements show a mean value of Ms ∼80 × 10^−3 ^A/m. Assuming that magnetite is the main remanence-bearing magnetic mineral (Ms = 4.8 × 10^5^ A/m), the mean concentration of magnetite in the core should be about 0.2 ppm.

#### High and low temperature measurements

An indication of the nature and composition of magnetic minerals can be investigated through the determination of their Curie temperature from the analysis of thermomagnetic curves (χ vs T °C). Out of seven studied specimens, only three samples from the highly magnetized top 30 cm of the core gave reliable, almost identical results. An example in Supplementary Fig. 4a shows the characteristic thermo-magnetic-curve of a specimen at 11 cm. The predominance of magnetite is confirmed by the maximum unblocking temperature near 580 °C.

RT-SIRM and ZFC curves are shown (see Supplementary Fig. 4b) for the sample at 60 cm. The ZFC curve clearly shows the Verwey transition at 120 K, which is characteristic of stoechiometric magnetite. The presence of siderite and pyrrhotite can be ruled out as the data contain no evidence for the 34 K transition in siderite or the 35 K transition in pyrrhotite^40,41^.

### Paleomagnetic record

**Figure 2:**
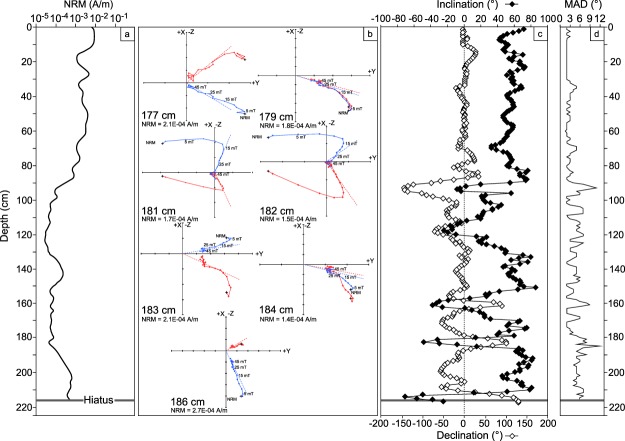
(**a**) NRM of BF2 core plotted from DAIE software. (**b**) Orthogonal vector diagrams during AF demagnetization of samples selected at the depth of a geomagnetic event at 181 cm. (**c**) Inclination and declination of BF2 core after deconvolution using the UDECON software. PCA were performed using DAEI software, using the Kirshwink method^31^. (**d**) Variation of MAD.

The BF2 core is almost pure calcite and the intensity of magnetization (M) is low. Natural remanent magnetization (NRM) shows relatively high values up to 9 × 10^−3 ^A/m from the top of the core to 20 cm (Fig. 2a). Intensity decreases gradually from 20 cm to 95 cm. At deeper levels, the magnetization ranges from 10^−4^ to ∼8 × 10^−4^ A/m. Osete *et al*.^19^ reported comparably high magnetization at the top of a stalagmite that stopped growing 126 ka ago. We selected seven end-point diagrams^42^ that describe a geomagnetic event recorded at 181 cm, dated to 521 ± 16/13 ka (Fig. 2b). The recorded magnetization is direct at depths 177 and 179 cm, reverse at depths 181, 182 and 183 cm, and direct again at depths 184 and 186 cm. All diagrams show secondary magnetization cleaned by 12–13 mT AF demagnetization. The Median Destructive Field (MDF) of NRM is in the 15–35 mT range, which is compatible with magnetite. After AF demagnetization and deconvolution, paleomagnetic inclination and declination show high synchronous variations with negative inclination, especially in the 216 cm to 90 cm range, which corresponds to the 537 ka to 416 ka time interval (Fig. 2c). Maximum angular deviation (MAD) values are large in weakly magnetized sections of the core where it does not exceed ∼12° (Fig. 2d).

## Discussion

**Figure 3:**
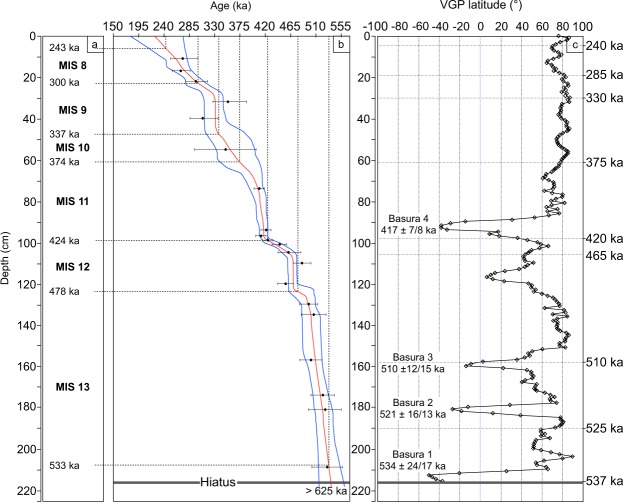
(**a**) MISs. (**b**) Consistency between the climates derived from MISs and the growth rates derived from the age model. The growth rate increases during interglacial periods and decreases during glacial periods. (**c**) Latitude of the VGP from 216 cm to the top of the core showing four geomagnetic excursions of the pole to southern latitude.

The paleomagnetic study of core BF2 revealed a record of several geomagnetic events during the Lower Brunhes period. The depth-age model estimates the age of these events and more generally the age of the calcitic growth history. Figure 3a displays ages and durations ranging from MIS 7 to MIS 13^32^. There is global consistency in Fig. 3b between the mean growth rate estimated from the age model and the MIS climate: the growth rate increases during interglacial periods and decreases during glacial periods. The top of the core is dated to 225 ka, in early MIS 7 (191–243 ka). During glacial MIS 8 (243–300 ka), the mean growth rate is low, ∼0.3 cm/ka. The mean deposition rate during the preceding interglacial stage, MIS 9, is relatively high, ∼0.7 cm/ka. Glacial MIS 10 shows a low mean growth rate of ∼0.4 cm/ka. The mean growth rate during interglacial MIS 11 is relatively high, ∼0.8 cm/ka, and, as expected, the mean growth rate during glacial MIS 12 is low: ∼0.5 cm/ka. The MIS 13 interglacial is marked by a high mean growth rate, ∼1.5 cm/ka. Calcite preserved a signal from past climates, in particular the consistency of higher growth rates during interglacials.

Requirements for reasonable RPI data for sediments are well documented^43,44^. The remanent vector used for RPI should be a single, well-defined component of magnetization. This is not the case for the BF2 core, as shown by the vector end-point Zijderveld diagrams (Fig. 2b). To our knowledge, RPI has not been widely used in speleothems. We propose bringing to light geomagnetic excursions using the virtual geomagnetic pole (VGP) latitude crossing the virtual geomagnetic equator (Fig. 3c).

Just after the hiatus, at 216 cm (dated to 536 ± 25/18 ka), the VGP latitude (Fig. 3c) shows a partly recorded excursion with negative values with a minimum of −50^◦^ at 213 cm, which we date to 534 ± 24/17 ka from the age model (Fig. 3b). We are calling this excursion Basura 1. There are several excursions with a similar age to that obtained for Basura 1. In the Sint 2000 stack, an excursion named ‘Calabrian’ is dated to 534 ka^45^. In their Table 1, Lund *et al*.^46^ propose an excursion that they call ‘14 A’, dated to 535 ka. Finally, in the GITS synopsis of Singer^3^, the excursion dated to 528–531 ka is referred to as ‘West Eifel 5′. We suggest that the age of the Basura 1 excursion is compatible with these three excursions described above.

A VGP excursion to negative latitude with a minimum of −63° is visible at 181 cm, which we date to 521 ± 16/13 ka (Fig. 3c). This excursion is called Basura 2. An evolution of the VGP latitude to −14^◦^ is centered at 160 cm, which we date to 510 ± 12/15 ka (Fig. 3c). We have named this excursion Basura 3. Several excursions have been described, but they cannot reasonably be linked to Basura 2 and 3. However, Langereis *et al*.^47^ propose an age of 515 ± 3 ka for an excursion called ‘Calabrian Ridge 2′. This excursion is considered to be poorly documented at 525 ka by Laj and Channell^4^ and considered to be possible by Roberts^2^. In their Table 1, Lund *et al*.^46^ propose an excursion named ‘13 A’, dated to 480–510 ka. This excursion is categorized as putative by Laj and Channel^4^. The high variability of the dates of these excursions could be explained by a two-fold excursion recorded by Basura 2 and 3.

An evolution of the VGP latitude to −38^◦^ is centered at 92 cm, which we date to 417 ± 7/8 ka. This excursion is named Basura 4. Langereis *et al*.^47^ found an ‘unnamed’ geomagnetic excursion dated in the 400–420 ka interval by sapropels in a core collected from the Calabrian Ridge. Lund *et al*.^46^ describe an excursion named ‘11 A’, dated to 420 ka in Ocean Drilling Program (ODP) site 1063 (Bermuda Rise). Channell *et al*.^48^ revisited the age model for the Brunhes Chron at ODP site 1063 using oxygen isotope and relative paleointensity data and documented a ‘Bermuda Rise’ excursion dated to 412 ka. In Chinese loess, Yang *et al*.^49^ brought to light an excursion ‘Baoji B’, dated to 422 ka. Oda^50^ describes a ‘Weinen’ excursion dated to 400–420 ka. The above mentioned excursions from different lithologies and sites, dated around 400–420 ka, appear to correspond to the same global excursion. We presume the Basura 4 excursion, with an age of 417 ± 7/8 ka, is related to these excursions.

Given the uncertainties in U-Th dating, the timing of the three distinct excursions, namely Basura 1, 2 and 3, overlaps. This made it difficult to unequivocally correlate the excursional behavior recorded in the speleothem by Basura 1, 2 and 3 with other well-documented excursions. Basura 4 is well dated to 417 ± 8 ka and its age is distinct from Basura 1, 2 and 3. Under ideal circumstances, but by pushing the limit of the precision of their U-Th dating method, Cheng *et al*.^17^ proposed reaching 2σ uncertainty of ± 6 ka at 500 ka, which corresponds to about 2.5 times less than the uncertainties displayed in this study. The excursion dated to 417 ± 7/8 ka should be considered as “possible” to refer to the classification^2,4^. In the near future, the expected progress in U-Th dating should result in the corroboration and the identification of the geomagnetic excursions Basura 1, 2 and 3 measured in this study. These excursions were all recorded at the same location, in a single flowstone, during interglacial MIS 11 and 13. Thouveny *et al*.^13^ observed the same result in sediment. In summary, this study confirms the potential of calcite speleothems for geomagnetic field records.

- The U-Th age model of the BF2 core shows that the mean growth rate is strongly dependent on the past climate. The core grew continuously from 536 ± 25/18 ka, from MIS 13 (533-478 ka) to MIS 8 (300-243 ka), and stopped growing at the beginning of MIS 7. A clear correlation was shown between the mean growth rate and glacial and interglacial MISs 13-8.
- Magnetic analyses showed that the main magnetic mineral is fine-grained magnetite in the range of pseudo-single-domain and single-domain grains.
- The paleomagnetism of the BF2 core, measured with the u-channel technique and deconvolved, shows four geomagnetic excursions were recorded in the core using VGP latitude and depth age model. Among them, one excursion occurred at 417 ± 7/8 ka was unambiguously identified.

In conclusion, this work shows that the unique properties of speleothems can help improve the Quaternary magnetostratigraphic scale.

## Supplementary information

Supplementary information
