# Supplementary material for: U-Th dated speleothem recorded geomagnetic excursions in the Lower Brunhes
Source: Sci Rep. 2019 Feb 4;9:1114. doi: 10.1038/s41598-018-38350-4 (PMC6361952; doi:10.1038/s41598-018-38350-4)
Supplement: Supplementary file 1 — Supplementary information [file 41598_2018_38350_MOESM1_ESM.pdf]

U-Th dated speleothem recorded geomagnetic excursions in the lower Brunhes

Jean-Pierre Pozzi, Louis Rousseau, Christophe Falguères, Geoffroy Mahieux, Pierre Deschamps, Qingfeng Shao, Djemâa Kachi, Jean-Jacques Bahain, and Carlo Tozzi

Supplementary information - Methods

U-series dating method

A first set of seven U-series dates were measured by thermal ionization mass spectrometry (TIMS) at the CEREGE (Aix-en-Provence, France) and GEOTOP (Montreal, Canada), using two distinct ^{236}U-^{233}U-^{229}Th mixed spikes. These spikes were calibrated following the procedure described by Deschamps[1]. Before total digestion in nitric acid, samples were spiked. U and Th were then co-precipitated with iron hydroxide, and then dissolved in nitric acid prior to chemical separation and purification using standard anion-exchange methods. This procedure employed U-TEVA resin (Eichrom) for uranium purification. Analyses of the BF2 flowstone were carried out during a preliminary stage of this study at GEOTOP. Measurements were performed using a VG sector TIMS fitted with an electrostatic filter and a Daly ion counter. The U and Th fractions were deposited on a single zone-refined Re filament between two layers of colloidal graphite. U and Th isotopes were measured in peak jumping mode on the Daly counter. Activity ratio and ages were calculated using the decay constants of Cheng[2]. Errors are at 2 σ with U-series ages calculated using the isoplot software[3]. Uranium concentrations are variable, ranging from 150 to 450 ppb. Thorium concentrations vary between 0.1 ppb and a few ppb. Although in most cases $^{230}Th/^{232}Th$ ratios are high, we implemented a correction of the detrital fraction by using the ^{232}Th concentration as an index of contamination[4]. We adopted the value of 1.7 established by Kaufman[5] for the ($^{230}Th/^{232}Th$) activity ratio in the detritus with a conservative uncertainty of ± 1.4 (2 σ). We assumed a secular equilibrium between ^{234}U and ^{238}U within the detrital fraction and a Th/U ratio similar to the terrestrial upper continental crust $(^{232}Th/^{238}U)_{AR} = 1.25 \pm 0.65$). Differences between corrected and non-corrected ages are low (< a few percent). Calculated initial $(^{234}U/^{238}U)_0$ ratios are relatively constant, with an average $(^{234}U/^{238}U)_0$ value of 1.143 ± 0.041 (2σ, n=14).

A second set of 14 U-series dates was performed at Nanjing Normal University (China). The procedures used for U/Th chemical separation and isotopic measurements are detailed in Shao[6]. The carbonate samples (50–150 mg) were weighed and dissolved in 7N HNO_3 in a Teflon beaker containing a known quantity of a ^{229}Th-^{233}U-^{236}U triple spike. The used $^{233}U/^{236}U$ spike is a gravimetric standard (CETAMA-MIRF2) having $^{233}U/^{236}U$ of 0.9683, which was gravimetrically calibrated using ultrapure U metal chips (CETAMA reference material MU2), and the $^{229}Th/^{233}U$ was calibrated with the HU-1 standard[7]. The sample-spike mixture was heated on a hot plate overnight to equilibrate. U and Th were preconcentrated by copricipitation with iron hydroxide and then separated from each other and from other cations by passing the sample solution through a U-TEVA resin column. The U/Th fractions were then dried and diluted in a mixture of 0.1N HNO_3 and 0.01N HF for isotopic analysis on a Neptune MC-ICPMS. U was measured statically by ^{233}U, ^{235}U, ^{236}U and ^{238}U on Faraday cups and ^{234}U simultaneously on a secondary electron multiplier (SEM). Th was analyzed with ^{230}Th and ^{229}Th alternately on the SEM and ^{232}Th on a Faraday cup, if it is > 3 mV (equivalent to 10^{11} Ω amplifier). In the case of ^{232}Th is < 3 mV, the ^{230}Th, ^{229}Th and ^{232}Th were measured on the SEM by peak jumping. The U isotopic ratios of the HU-1 standard were measured before and after every U and Th isotopic measurement for samples. Mass fractionation was corrected by comparing the measured $^{238}U/^{235}U$ to the natural value of 137.760 for HU-1 and 137.818 for unknown samples[8]. The SEM to Faraday cup yield was assessed by the $\delta^{234}U$ measured in the HU-1 standard. Hydride interferences, machine abundance sensitivity and amplifier gains were evaluated every day prior to sample measurements. $^{230}Th/U$ ages were calculated using half-lives of 75,584 years and 245,620 years for ^{230}Th and ^{234}U, respectively[2]. The $^{230}Th/U$ age uncertainty was estimated by Monte-Carlo simulations ($n=10^6$) with consideration of most sources of analytical uncertainty. Corrected ^{230}Th ages assume an initial $^{230}Th/^{232}Th$ activity ratio of 1.7±1.4 (2σ), as mentioned above.

1. Deschamps, P. *et al*. Further investigations on optimized tail correction and high-precision measurement of uranium isotopic ratios using multi-collector ICP-MS. *Chem. Geol.* **201**, 141-160 (2003).
2. Cheng, H. *et al*. Improvements in 230Th dating, 230Th and 234U half-life values, and U/Th isotopic measurements by multi-collector inductively coupled plasma mass spectrometry: *Earth and Planet. Sci. Lett.* **371-372**, 82-91 (2013).
3. Ludwig, K.R. Isoplot/Ex version 3.00. A Geochronological Toolkit for Microsoft Excel, User's Manual. *Berkeley Geochron. Ctr. Spec. Pub.* **4**: 1-70 (2003).
4. Dorale, J.A., *et al*. Uranium-series dating of speleothems: current techniques, limits and applications. In Sasowska, I. & Mylroie, J. (eds.) *Studies of Caves Sediments*, 177-197 (Springer US, 2004).
5. Kaufman, A. 1993. An evaluation of several methods for determining 230Th/U ages in impure carbonates. *Geochim. Cosmochim. Acta*. **57**, 2303-2317.
6. Shao, Q.F. et al. High precision U/Th dating of the rock paintings at Mt. Huashan, Guangxi, southern China. *Quatern. Res.* **88**, 1-13 (2017).
7. Frank N. *et al*. Open system U-series ages of corals from a subsiding reef in New Caledonia: Implications for sea level changes, and subsidence rate. *Earth and Planet. Sci. Lett.* **249**, 274–289 (2006).
8. Hiess, J. *et al*. 238U/235U systematics in terrestrial uranium-bearing minerals. *Science*. **335**, 1610-1614 (2012).

U-Th dated speleothem recorded geomagnetic excursions in the lower Brunhes

Jean-Pierre Pozzi, Louis Rousseau, Christophe Falguères, Geoffroy Mahieux, Pierre Deschamps, Qingfeng Shao, Djemâa Kachi, Jean-Jacques Bahain, and Carlo Tozzi

Supplementary Table S1

Table 1 ^{230}Th/U dating results for the BF2 samples

Sample ID	Depth[a] (cm)	^{238}U (ppb)	^{232}Th (ppb)	[^{234}U/^{238}U][d,e]	[^{230}Th/^{238}U][d,e]	[^{230}Th/^{232}Th][d,e]	Uncorrected age[e] (ka, BP)	[^{234}U/^{238}U]$_{initial}$[d,e] (corrected)	Corrected age[e,f] (ka, BP)
BF2-11[b]	11	173.8 ± 0.8	3.74 ± 0.02	1.0830 ± 0.0078	1.0400 ± 0.0114	148 ± 2	303 ± 23/19	1.195 ± 0.014	273 ± 27/21
BF2-17[b]	17	429.4 ± 1.8	3.87 ± 0.03	1.0708 ± 0.0077	1.0015 ± 0.0103	340 ± 4	272 ± 17/14	1.153 ± 0.013	269 ± 17/15
BF2-22[b]	22	268.5 ± 1.2	3.06 ± 0.02	1.0581 ± 0.0067	1.0082 ± 0.0085	272 ± 3	301 ± 19/16	1.136 ± 0.012	296 ± 21/18
BF2-32[b]	32	293.3 ± 1.3	0.79 ± 0.01	1.0518 ± 0.0068	1.0281 ± 0.0085	1175 ± 10	355 ± 34/26	1.141 ± 0.013	353 ± 33/26
BF2-40[b]	40	247.4 ± 1.1	6.08 ± 0.04	1.0577 ± 0.0043	1.0199 ± 0.0085	127 ± 1	322 ± 19/16	1.143 ± 0.009	308 ± 28/22
BF2-55[b]	55	291.0 ± 1.3	1.79 ± 0.01	1.0354 ± 0.0098	1.0173 ± 0.0102	502 ± 6	386 ± 55	1.105 ± 0.019	349 ± 55
BF2-105[b]	105	307.8 ± 0.5	0.44 ± 0.01	1.0424 ± 0.0016	1.0438 ± 0.0020	2255 ± 3	467 ± 22/18	1.157 ± 0.006	461± 22/18
BF2-74[c]	74	340.1 ± 0.1	6.34 ± 0.01	1.0346 ± 0.0008	1.0230 ± 0.0018	168 ± 1	410 ± 9	1.110 ± 0.004	409 ± 9
BF2-94[c]	94	406.4 ± 0.1	2.59 ± 0.01	1.0293 ± 0.0009	1.0184 ± 0.0015	488 ± 1	421 ± 9	1.096 ± 0.004	421 ± 9
BF2-97[c]	97	314.5 ± 0.1	4.52 ± 0.01	1.0358 ± 0.0010	1.0252 ± 0.0016	218 ± 1	413 ± 9	1.114 ± 0.004	412 ± 9
BF2-99[c]	99	237.3 ± 0.1	5.13 ±0.01	1.0348 ± 0.0007	1.0266 ± 0.0016	145 ± 1	425 ± 9	1.115 ± 0.004	424 ± 9
BF2-101[c]	101	280.9 ± 0.2	0.43 ± 0.01	1.0462 ± 0.0012	1.0459 ± 0.0016	2107 ± 22	445 ± 13	1.162 ± 0.007	445 ± 13
BF2-110[c]	110	387.3 ± 0.1	0.11 ± 0.01	1.0314 ± 0.0009	1.0315 ± 0.0014	11336 ± 659	485 ± 16	1.123 ± 0.007	485 ± 16
BF2-120[c]	120	419.1 ± 0.3	0.24 ± 0.01	1.0338 ± 0.0012	1.0308 ± 0.0016	5550 ± 127	456 ± 14	1.122 ± 0.006	456 ± 14
BF2-130[c]	130	413.0 ± 0.1	0.08 ± 0.01	1.0304 ± 0.0009	1.0316 ± 0.0013	15885 ± 782	497 ± 16	1.124 ± 0.007	497 ± 16
BF2-135[c]	135	268.8 ± 0.1	0.04 ± 0.01	1.0339 ± 0.0011	1.0373 ± 0.0016	21645 ± 2693	506 ± 22	1.141 ± 0.010	506 ± 22
BF2-157[c]	157	245.1 ± 0.1	0.02 ± 0.01	1.0361 ± 0.0009	1.0398 ± 0.0017	76386 ± 36691	501 ± 20	1.148 ± 0.009	501 ± 20
BF2-174[c]	174	273.6 ± 0.1	0.02 ± 0.01	1.0340 ± 0.0008	1.0389 ± 0.0015	298800 ± 53212	522 ± 21	1.148 ± 0.009	522 ± 21
BF2-181[c]	181	215.5 ± 0.1	0.02 ± 0.01	1.0256 ± 0.0011	1.0274 ± 0.0018	38028 ± 11458	523 ± 32/26	1.113 ± 0.010	523 ± 32/26
BF2-209[c]	209	235.0 ± 0.1	0.77 ± 0.01	1.0278 ± 0.0010	1.0310 ± 0.0016	958 ± 7	530 ± 27	1.124 ± 0.010	530 ± 27
BF2-219[c]	219	218.6 ± 0.1	0.76 ± 0.01	1.0576 ± 0.0009	1.0814 ± 0.0016	952 ± 5	> 625	-	-

Analytical errors are 2σ of the mean.

[a] Distance from top of the BF2 core.

[b] ^{230}Th/U dating results by TIMS (CEREGE, France; GEOTOP, Canada).

[c] ^{230}Th/U dating results by MC-ICPMS (Nanjing Normal University, China).

[d] Square brackets denote activity ratios.

[e] Activity ratios and ^{230}Th/U ages were calculated with the decay constants of Jaffey et al., 1971 (^{238}U), Cheng et al., 2013 (^{234}U and ^{230}Th) and Holden, 1990 (^{232}Th).

[f] Dates reported as ka Before Present (BP), where the Present is defined as the year 1950 CE.

1. Jaffrey, A.H.. *et al*. Precision measurement of half-lives and specific activities of 235U and 238 U. *Phys. Rev. C* **4**, 1889 (1971).
2. Cheng, H. *et al*. Improvements in 230Th dating, 230Th and 234U half-life values, and U/Th isotopic measurements by multi-collector inductively coupled plasma mass spectrometry: *Earth and Planet. Sci. Lett*. **371-372**, 82-91 (2013).
3. Holden, N.E. Total half-lives for selected nuclides. *Pure Appl. Chem*. **62**:5, 941-958 (1990).

Supplementary Fig. S2

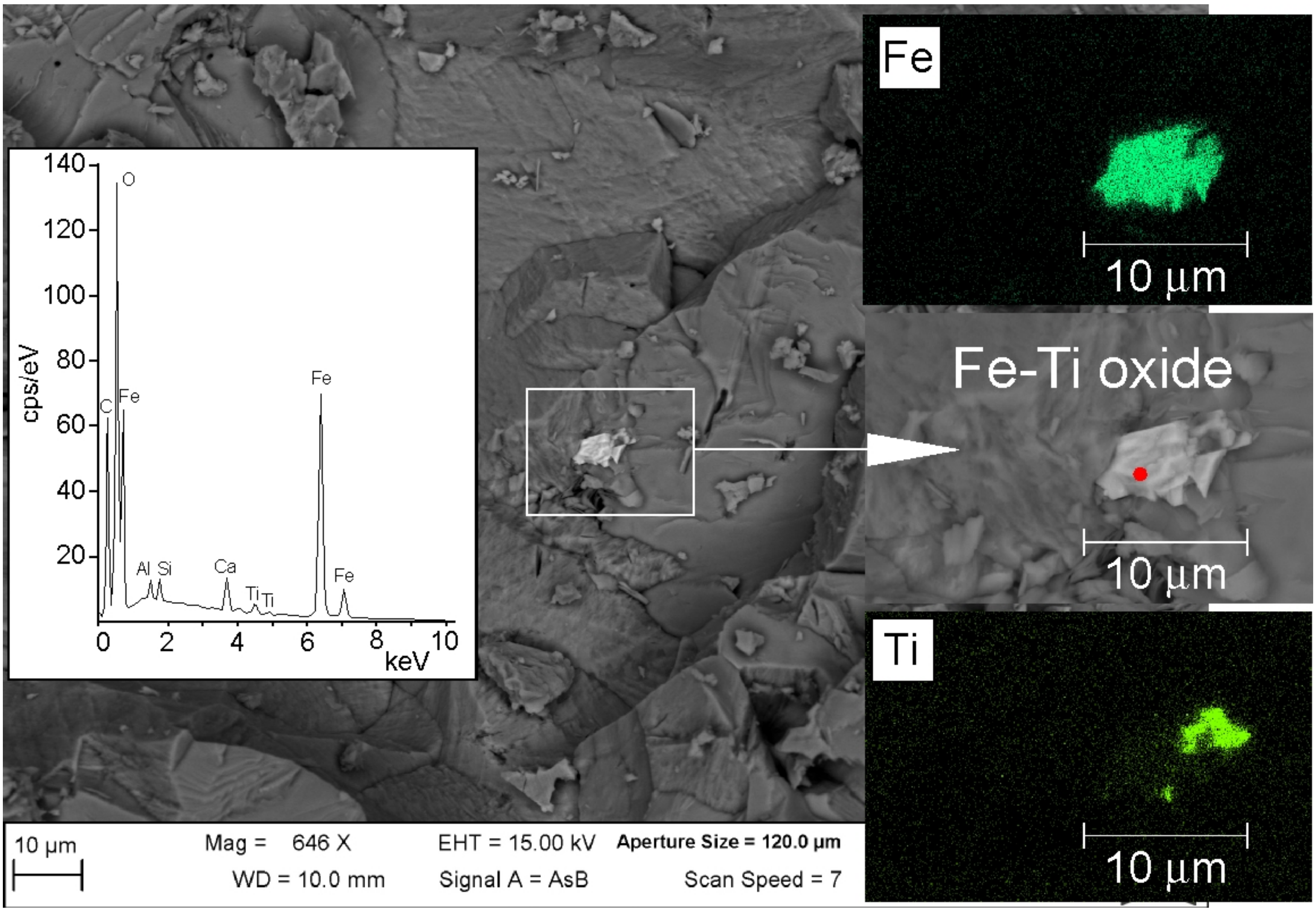

Scanning electron microscopy from fresh rock fragments of BF2 core. Composition mapping and EDS spectra of detrital Fe-Ti oxide.

<u>Supplementary Fig. S3</u>

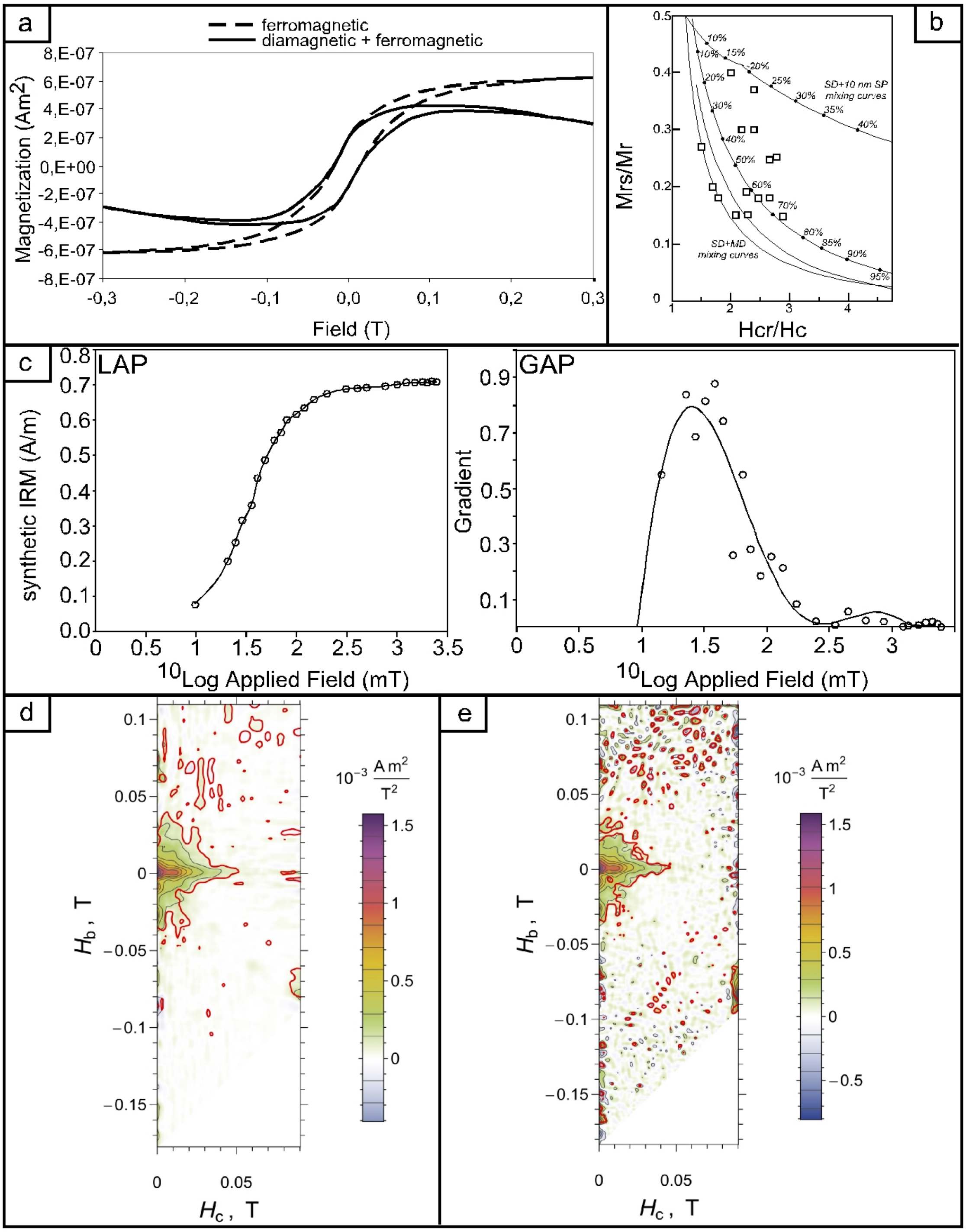

a- Example of hysteresis cycle achieved with a sample located at 52 cm in BF2 core. The calcite diamagnetism dominates the signal in higher field. When removed, the ferromagnetic fraction shows saturation near 0.3 T compatible with magnetite. Magnetization is measured up to 2.2 T, but it is represented up to 0.3 T for clarity. b- Hysteresis data plotted on the theoretical unmixing Dunlop diagram and showing PSD + SD trend. c- Isothermal remanent magnetization (IRM) acquisition curves after treatment by the cumulative log-Gaussian function. Example of linear acquisition plot (LAP) and gradient acquisition plot (GAP) showing a bimodal association of coercivity spectra. d- First order reversal curve (FORC) diagram plotted with a variable smoothing. e- FORC diagram plotted with a fixed smoothing factor (SF = 3). PSD + SD

Supplementary Fig. S4

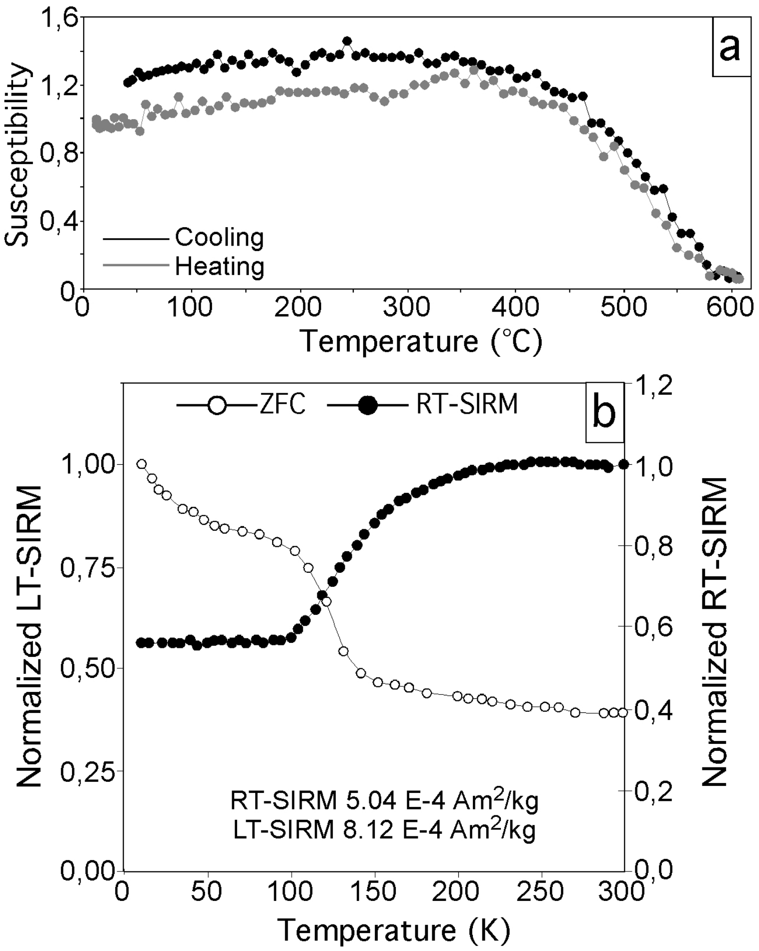

a- Thermomagnetic curves of a specimen sampled at 11 cm, in the highly magnetized upper part of the core, and heated in controlled argon-atmosphere. Heating and cooling curves show that mineralogical transformations of magnetic minerals by heating are not very important.

b- Low temperature analyses. RT-SIRM: 2.5 T saturation IRM (SIRM) is imparted at room temperature (RT) and the specimen cooled from 300 K to 10 K. LT-SIRM: 2.5 T SIRM is imparted at 10 K (LT) after zero-field cooling (ZFC) and the specimen is thermally demagnetized by warming to 300 K. The ZFC curve clearly shows the Verwey transition at 120 K.
